# FAIMS Enhances the Detection of PTM Crosstalk Sites

**DOI:** 10.1021/acs.jproteome.1c00721

**Published:** 2022-03-02

**Authors:** Kish R. Adoni, Debbie L. Cunningham, John K. Heath, Aneika C. Leney

**Affiliations:** School of Biosciences, University of Birmingham, Edgbaston, Birmingham B15 2TT, U.K.

**Keywords:** post-translational modifications, crosstalk, FAIMS, proteomics, mass spectrometry

## Abstract

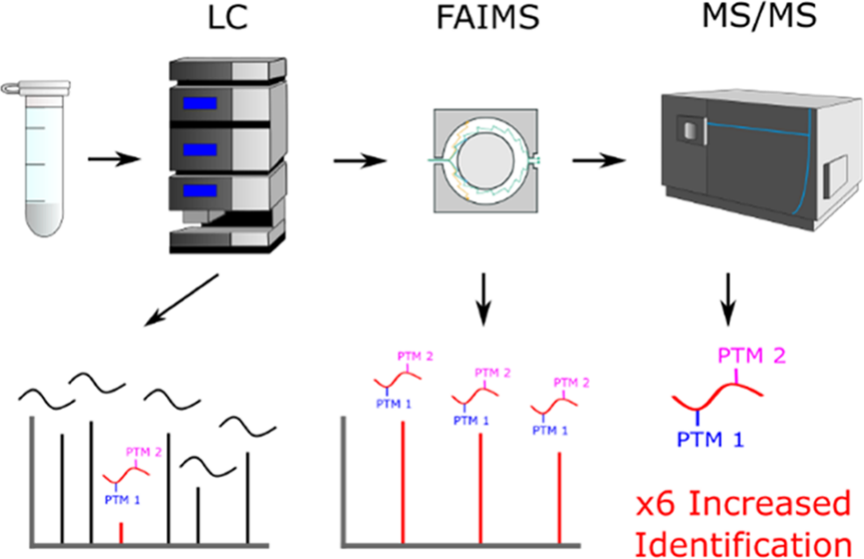

Protein post-translational modifications (PTMs) enable cells to rapidly change in response to biological stimuli. With hundreds of different PTMs, understanding these control mechanisms is complex. To date, efforts have focused on investigating the effect of a single PTM on protein function. Yet, many proteins contain multiple PTMs. Moreover, one PTM can alter the prevalence of another, a phenomenon termed PTM crosstalk. Understanding PTM crosstalk is critical; however, its detection is challenging since PTMs occur substoichiometrically. Here, we develop an enrichment-free, label-free proteomics method that utilizes high-field asymmetric ion mobility spectrometry (FAIMS) to enhance the detection of PTM crosstalk. We show that by searching for multiple combinations of dynamic PTMs on peptide sequences, a 6-fold increase in candidate PTM crosstalk sites is identified compared with that of standard liquid chromatography-tandem mass spectrometry (LC-MS/MS) workflows. Additionally, by cycling through FAIMS compensation voltages within a single LC-FAIMS-MS/MS run, we show that our LC-FAIMS-MS/MS workflow can increase multi-PTM-containing peptide identifications without additional increases in run times. With 159 novel candidate crosstalk sites identified, we envisage LC-FAIMS-MS/MS to play an important role in expanding the repertoire of multi-PTM identifications. Moreover, it is only by detecting PTM crosstalk that we can “see” the full picture of how proteins are regulated.

## Introduction

Dynamic modifications impart order on proteins enabling them to adapt their function and respond to a changing environment. Enzyme-mediated post-translational modification (PTM) enables proteins to change their location, function, or abundance in response to stimuli. With over 450 PTMs reported,^1^ the framework for specialized responses of proteins to their cellular environment is laid. Each of these PTMs impacts the function of proteins in one way or another. Moreover, many proteins exhibit multiple PTMs that differentially contribute to protein function.^2,3^ One PTM can also regulate the occurrence of another PTM on the same protein, a phenomenon known as PTM crosstalk or PTM interplay.^2,4^ PTM crosstalk between phosphorylation and O-GlcNAcylation was the first reported.^5^ Due to shared modification of the hydroxyl group of serine and threonine residues, reciprocal crosstalk (whereby one modification competes with the other on the same site), positive crosstalk (whereby one modification enhances the modification on a site within the same protein), and negative crosstalk (whereby one modification inhibits the modification of another site) can occur.^5,6^ PTM crosstalk between many different PTMs, including acetylation and phosphorylation,^7^ ubiquitination and phosphorylation,^8,9^ acetylation and methylation,^10^ and methylation and phosphorylation^11^ is also observed. A prominent example of PTM crosstalk involves histones; here, multiple PTMs such as methylation, acetylation, and ubiquitination interact to regulate the epigenome,^12^ showing that further understanding the mechanisms of PTM crosstalk will inform understanding of various biological processes. Indeed, it is becoming increasingly apparent that the interplay between PTMs in neurological diseases,^13^ diabetes,^14^ and cancer^15^ can be dysregulated, contributing to disease pathology.^16^

The first step in deciphering the role of PTM crosstalk is detecting which proteins are post-translationally modified and whether PTM crosstalk occurs. Traditionally, analysis at the cellular level involves upregulating one PTM (by adding an enzyme inhibitor/activator) and globally monitoring another via western blotting^17^ or quantitative proteomics.^2^ This approach does not reveal the specific post-translationally modified amino acid residues linked to PTM crosstalk, although thousands of crosstalk sites are identified. Other work has systematically looked at the co-occurrence of PTMs on synthetic peptides from within proteins with the view to predict or even rule out sequence motifs that may be involved in PTM crosstalk.^18−20^ However, this does not reflect proteins in their physiological environment. Thus, an enhanced methodology for detecting PTM crosstalk in vivo is needed.

Shotgun proteomics is rapidly accelerating in its ability to detect PTMs owing to its high-throughput nature and simultaneous ability to site-localize PTMs.^21,22^ Due to its ability to detect multi-PTM-containing peptides in an unbiased manner, this technique has great potential for detecting putative positive PTM crosstalk sites that cellular assays could further validate.^23^ There are limitations, however, since proteins are modified substoichiometrically, and thus, the highly abundant unmodified peptides are identified at the expense of their PTM-containing counterparts. When searching for positive PTM crosstalk, identifying multiple-PTM sites on the same peptide region within a protein exacerbates the problem. PTM enrichment strategies have been introduced to address this issue and used to search for PTM crosstalk sites from which crosstalk motifs can be deciphered.^24^ However, not all PTMs have a viable enrichment technique, and enrichment techniques that rely on the use of PTM specific antibodies which have been tailored to a specific antigen can be biased when looking at these modifications at the proteome level. Thus, we sought to develop unbiased shotgun proteomics methods to detect positive PTM crosstalk. In this regard, the inclusion of an online ion mobility device after LC separation and before MS/MS analysis has shown promise. Coon and co-workers have shown that with the addition of a high-field asymmetric ion mobility spectrometry (FAIMS) device, protein and phosphosite identifications dramatically increase.^25,26^ Also, increased numbers of multiphosphorylated peptides have been observed.^27^ Moreover, ion mobility spectrometry is advantageous in separating peptide isomers, whereby both peptides contain the same PTM but at different sites.^28^

Here, we show by incorporating an online FAIMS device into an automated LC-MS/MS workflow, without prior protein/peptide PTM enrichment, that the number of multi-PTM-containing peptides identified increases. Strikingly, 40% of these multi-PTM peptides have not previously been detected, highlighting the advantages of incorporating FAIMS to identify novel candidate positive PTM crosstalk sites. Additionally, we show that through optimization of the FAIMS parameters, LC-FAIMS-MS/MS can enhance the detection of candidate PTM crosstalk sites on the same timeframe as a standard LC-MS/MS workflow. With PTM crosstalk now appreciated as a widespread mechanism of protein signaling, incorporating this workflow in routine proteomics analysis will help gain insight into the biochemistry of signaling pathways across the human proteome and improve our mechanistic understanding of aberrant PTM crosstalk in disease.

## Methods

### Chemicals and Reagents

All materials were purchased from Thermo Fisher Scientific unless otherwise stated. Pierce HeLa Protein Digest Standard was used throughout. The HeLa digest was reconstituted in 10% (v/v) formic acid at a concentration of 200 ng/μL and stored at −20 °C prior to use. One microgram of HeLa digest was used for all experiments.

### Liquid Chromatography

Online nanoliquid chromatography (nanoLC) was performed using UltiMate 3000 RSLCnano system with an Acclaim PepMap 100 Å C18 3 μm 75 μm × 500 mm analytical column and Acclaim PepMap 100 Å C18 3 μm 0.075 × 20 mm^2^ trap cartridge (Thermo Fisher Scientific). Peptides were separated using a gradient with mobile phases A (100% H_2_O, 0.1% HCOOH) and B (100% ACN, 0.1% HCOOH) and eluted using a gradient from 3.2 to 44% B over 155 min. The column flow rate was set to 350 nL/min with column temperature set at 40 °C.

### Field Asymmetric Ion Mobility Spectrometry

The FAIMS Pro source (Thermo Fisher Scientific) was located between the nanoESI source and the mass spectrometer. Parameters for the FAIMS device were as follows: inner electrode temperature, 100 °C; outer electrode temperature, 100 °C; carrier gas flow rate, 0 L/min; asymmetric waveform with dispersion voltage, −5000 V; and entrance plate voltage, 250 V. N_2_ was used as the FAIMS carrier gas and the FAIMS Pro ion separation gap was 1.50 mm. For static CV conditions, the selected CV (0, −45, −52.5, −60, −67.5, −75, and −90 V) was applied throughout the LC-MS/MS run. To perform the internal stepping, the FAIMS device was set to cycle between −45, −60, −75, and −90 V each for 0.75 s before performing the next MS1 scan. Control conditions were run with the FAIMS source detached from the instrument. All conditions were run in triplicate.

### Mass Spectrometry

The nanoLC system was coupled to an Orbitrap Eclipse Tribrid Mass Spectrometer (Thermo Fisher Scientific). A SilicaTip (FS360-75-15-N) was used, and the voltage was applied through an HPLC liquid junction tee between the column and the tip. The spray voltage was optimized for each SilicaTip. The instrument was operated with Instrument Control Software version 3.3.2782.34. The Eclipse was externally calibrated using Pierce FlexMix Calibration Solution (Thermo Fisher Scientific). Instrument parameters were as follows: transfer capillary temperature 300 °C and RF lens at 30%. MS1 spectra were acquired in the Orbitrap analyzer using a resolution of 60 K, automatic gain control (AGC) target: 100%, maximum injection time of 50 ms, and a mass range between 380 and 2000 *m*/*z*. MS1 spectra were recorded at 3 s intervals unless otherwise stated. For MS/MS experiments, precursor ions were isolated in the quadrupole with a 1.2 *m*/*z* window, monoisotopic precursor selection (MIPS) was turned on, and precursor ions were subjected to higher-energy collision dissociation (HCD) with a normalized HCD energy of 35%. Dynamic exclusion was employed for 60 s on a single-charge state per precursor, and only charge states from 3+ to 8+ were selected for MS/MS. Filter precursor priority for MS/MS analysis was based on the highest charge state due to the high frequency of missed cleavages observed in PTM-containing peptides. MS/MS spectra were acquired in the ion trap mass analyzer with the scan rate set to “Rapid,” scan range set to “auto,” and maximum injection time to “dynamic.”

### Data Analysis

All RAW files were processed and analyzed using Thermo Proteome Discoverer v.2.4. (Thermo Fisher Scientific). All database searches were performed using PMi-Byonic.^29^ The default settings were predominantly used in Proteome Discoverer unless otherwise stated. Homo sapiens (SwissProt TaxID 9606, 20 444 proteins) generated September 2019 was used for the protein database when two different dynamic PTMs were searched. For the broad PTM search, a bespoke FASTA file was created, which included only the proteins that were detected without standard PTMs in two out of three replicates of a FAIMS condition or standard LC-MS. For all searches, trypsin cleavage was set to full, and the maximum number of missed cleavages set to 4. A precursor mass tolerance of 10 ppm was used, with a fragment mass tolerance of 0.5 Da. The raw data was searched twice, first for combinations of two different, dynamic PTMs on peptides and second for a wider range of PTM combinations that could be found on proteins. When searching data for two different, dynamic PTMs, the static modifications included carbamidomethylation (+57.021 Da) of cysteine and dynamic modifications included oxidation (+15.995 Da) of methionine, N-terminal methionine loss (−131.040 Da), and N-terminal methionine loss plus acetylation (−89.030 Da). The additional pairwise combinations that were included in the search were phosphorylation (+79.996 Da; S, T, Y), acetylation (+42.011; K, R, N-terminal), and methylation (+14.016; K, R). For example, for phosphorylation–acetylation crosstalk, we included phosphorylation (+79.996 Da; S, T, Y) and acetylation (+42.011; K, R) within the search but no other dynamic modifications except for the common aforementioned dynamic modifications. Moreover, by searching pairwise for PTM combinations rather than “open” searches, the computational requirements for database searching are minimized, taking ∼8 h per technical replicate for all FAIMS parameters included in this study. For the wider PTM search, the additional PTMs that were investigated included the following: phosphorylation (+79.996 Da; S, T, Y), acetylation (+42.011; K, R), methylation (+14.016; K, R), nitrosylation (+28.990; C, Y), deamidation (+0.984; N), GG corresponding to ubiquitination (+114.042; K), and HexNAc (+203.079; N, S, T). Note that in all cases, the N-terminal methionine loss plus acetylation dynamic modification (−89.030; protein N-terminus) was included as part of the “Acetyl” PTM. The total common modifications for all searches was set to 4. Percolator node was set to filter for an FDR of 0.01 and 0.05 with a final filtering set of 0.01 applied in the final Proteome Discoverer results file. For the PTM-wide search, the Byonic score threshold for PSM identifications is set to ≥ 300 to minimize false-positive identifications. For PTM site localization, ptmRS was used.^30^ For pairwise PTM searches, the PTM site probability threshold was set to 75. For the PTM wide search, the site probability threshold was set to 100 for at least one PTM with the other at ≥75.

To additionally validate that our identifications reported were not entirely false positives, we added 200 randomly chosen sequences of lengths ranging from 6 to 30 amino acids to our protein database. None of these sequences were present in Byonic searches using all RAW files generated with and without FAIMS, validating that the data we are reporting is highly unlikely to be entirely false-positive identifications. For data processing, a 64 GB Intel(R) Core(TM) i9-10900 CPU @ 2.80 GHz with 12 virtual cores was used with Windows 10 Enterprise N. A peptide that contained two or more of PTMs such as phosphorylation, acetylation (including methionine loss plus acetylation at N-terminus), methylation, deamidation, nitrosylation, ubiquitination, and glycosylation (HexNAc) was defined as a “multi-PTM peptide.” Peptides of differing lengths or of the same length but with additional “unnatural” modifications (carbamidomethylation and methionine oxidation) to the ones detected were treated as one candidate positive PTM crosstalk site. The multi-PTM peptides whereby the mass difference of the two different PTMs is equivalent were excluded from the data set. In all cases, peptides that were identified in at least two replicates of a single condition were reported. A summary table of all multi-PTM-containing peptides identified in two out of three LC-(FAIMS)-MS/MS conditions along with their PTM site probabilities is provided in the Supporting Information. In the cases whereby multi-PTM-containing peptides were present with and without additional non-natural modifications, both peptides were included in the associated data file. Likewise, when two multi-PTM-containing peptides with different sequence lengths were observed within peptide groups, these were reported, however, were treated as one candidate PTM crosstalk site.

## Results and Discussion

### LC-FAIMS-MS/MS Detects Candidate Crosstalk Sites

**Figure 1:**
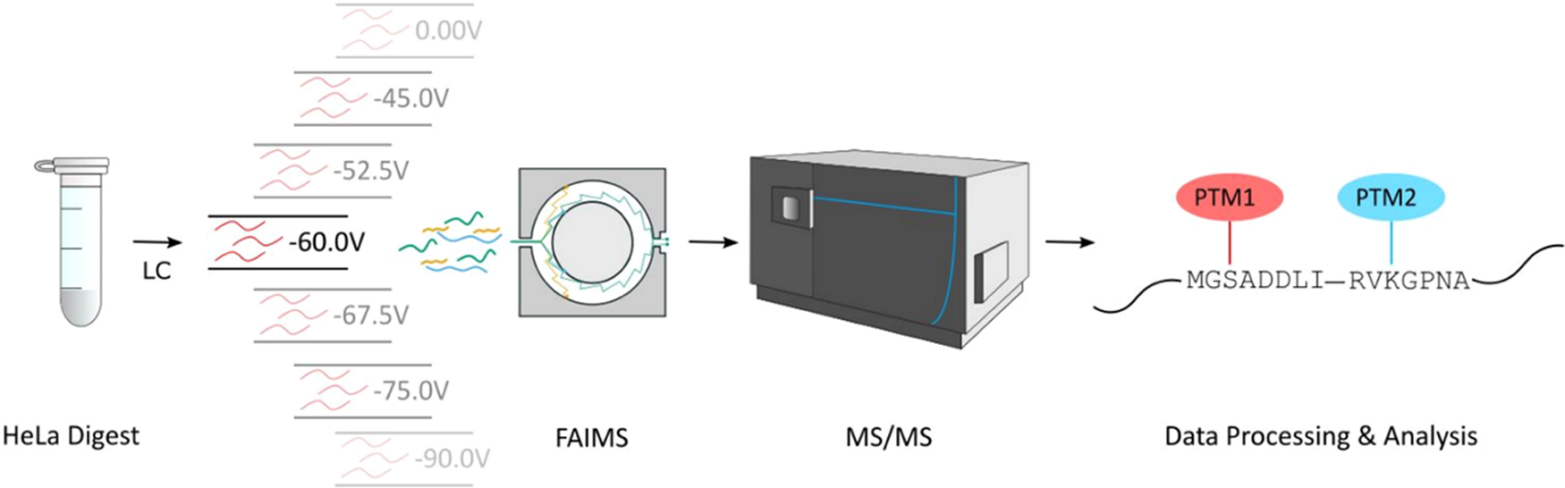
Experimental workflow. Peptides from a HeLa digest were subjected to online reverse-phase liquid chromatography (LC) before online gas-phase separation using high-field asymmetric ion mobility spectrometry (FAIMS) and mass spectrometry (MS) analysis. The MS data was searched in Proteome Discoverer for any multi-PTM peptides that were detected, the identified “hits” being potential PTM crosstalk site candidates.

**Figure 2:**
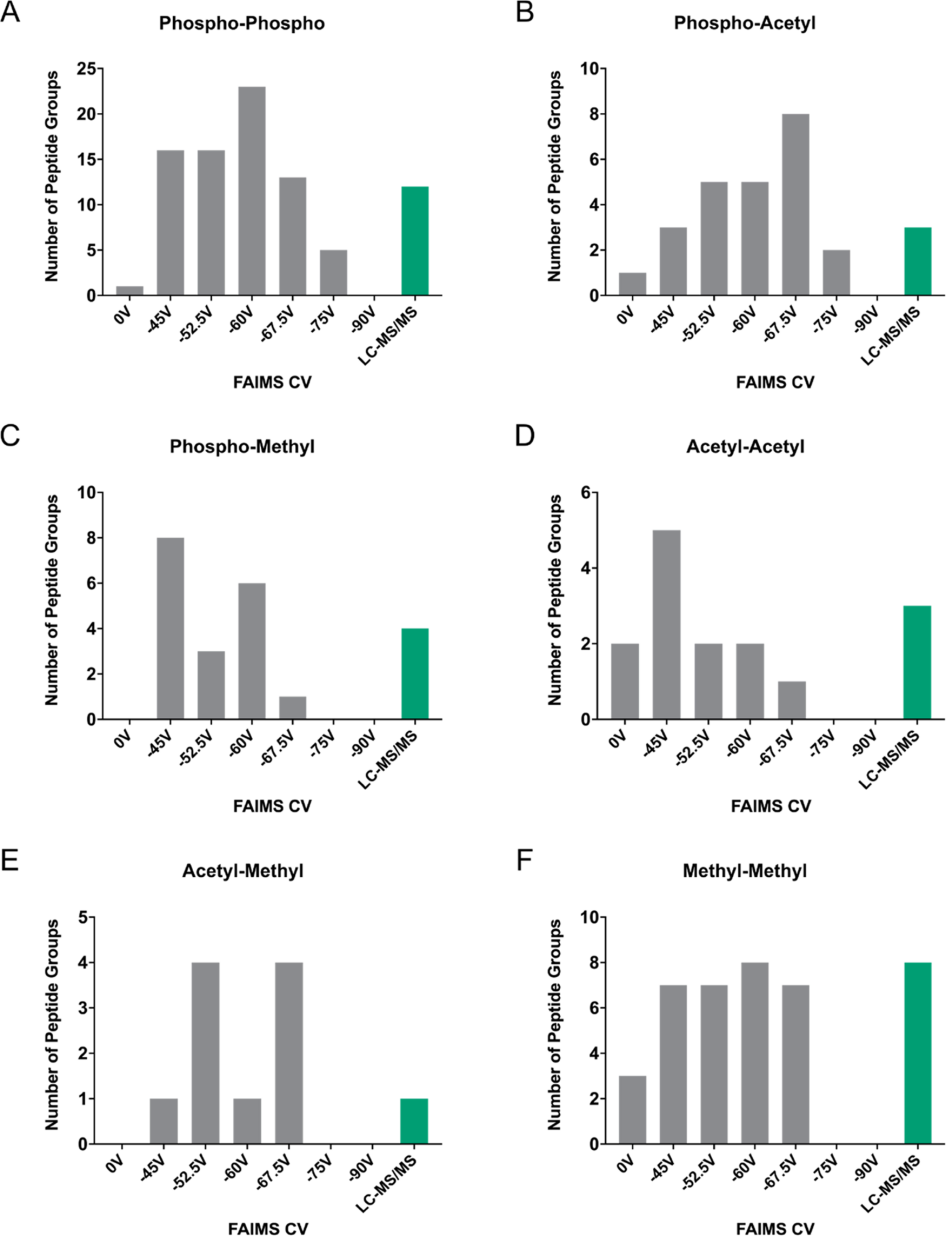
FAIMS improves identification of PTM crosstalk sites. The number of peptide groups in which multi-PTM peptides were observed as a function of FAIMS compensation voltage (CV) for the PTM combinations: multiphosphorylation (phospho–phospho) (A), phosphorylation–acetylation (phospho–acetyl) (B), phosphorylation–methylation (phospho–methyl) (C), multiacetylation (acetyl–acetyl) (D), acetylation–methylation (acetyl–methyl) (E), and multi-methylation (methyl–methyl) (F). The number of peptide groups detected with the FAIMS source removed from the instrument (LC-MS/MS) is included in each case.

Due to the low stoichiometry of protein PTMs and the amplification of this problem in the context of PTM crosstalk, we sought to set up an online separation strategy, whereby post-LC separation, FAIMS was used to selectively transfer peptides into the mass spectrometer for MS/MS analysis (Figure 1). We hypothesized that FAIMS would separate peptides in an orthogonal manner compared with that of LC separation, thus simplifying the MS1 spectra and making multi-PTM peptides more likely to be selected for MS/MS analysis. To exemplify our strategy, tryptic peptides from a human cervix carcinoma cell line, HeLa S3, were used. Due to the varying properties of the tryptic peptides, LC-MS/MS replicates were performed using a range of static compensation voltages (0, −45.0, −52.5, −60.0, −67.5, −75.0, and −90.0 V) and the sum of the different peptides identified at each compensation voltage was compared to the number of peptides identified when the FAIMS source was removed from the instrument (Figure 2). Consistent with previous work by Coon and co-workers,^26^ we report an increase in the number of overall peptide and protein identifications when comparing the sum of all single compensation voltages (hereafter termed external stepping) to standard LC-MS/MS without FAIMS (Figure S1), showing the power of FAIMS in enhancing proteome coverage.

Next, we sought to investigate the effect of applying external stepping on the detection of multi-PTM-containing peptides. Indeed, positive PTM crosstalk can be inferred if peptides are detected from the same protein harboring two or more PTMs. Thus, to seek out candidate positive PTM crosstalk sites, the theoretical database (upon which the raw MS/MS spectra were compared to) was expanded to include peptides harboring a minimum of two of the following dynamic PTMs: S/T/Y phosphorylation, R/K/N-terminal acetylation, and R/K methylation, and the raw data searched sequentially against these larger databases. As predicted, based on their differential properties and thus differential ion mobility, LC-FAIMS-MS/MS vastly improved the number of multi-PTM peptide identifications for all PTM combinations (Figure 2). Moreover, even when a single compensation voltage was applied, in some cases, the number of candidate PTM crosstalk sites detected was greater than those detected by LC-MS/MS alone. Although some trends are apparent between the static compensation voltages used and the number of different multi-PTM-containing peptides identified, the reasoning for this is yet to become apparent. Moreover, the optimum FAIMS compensation voltage likely depends more on the protein sequence than the chemical composition of the PTMs added. Overall, 180 multi-PTM peptides corresponding to 158 proteins were reported from these LC-FAIMS-MS/MS-based experiments, representing a 6-fold increase in multi-PTM peptide identifications compared to that of standard LC-MS/MS (Figure S3). Excitingly, 51 of these are novel candidate positive PTM crosstalk sites that have not previously been reported in iPTMnet,^31^ PhosphoSitePlus,^32^ or Proteomics DB.^33^

### FAIMS Internal Stepping Accelerates Identification of Candidate Crosstalk Sites

**Figure 3:**
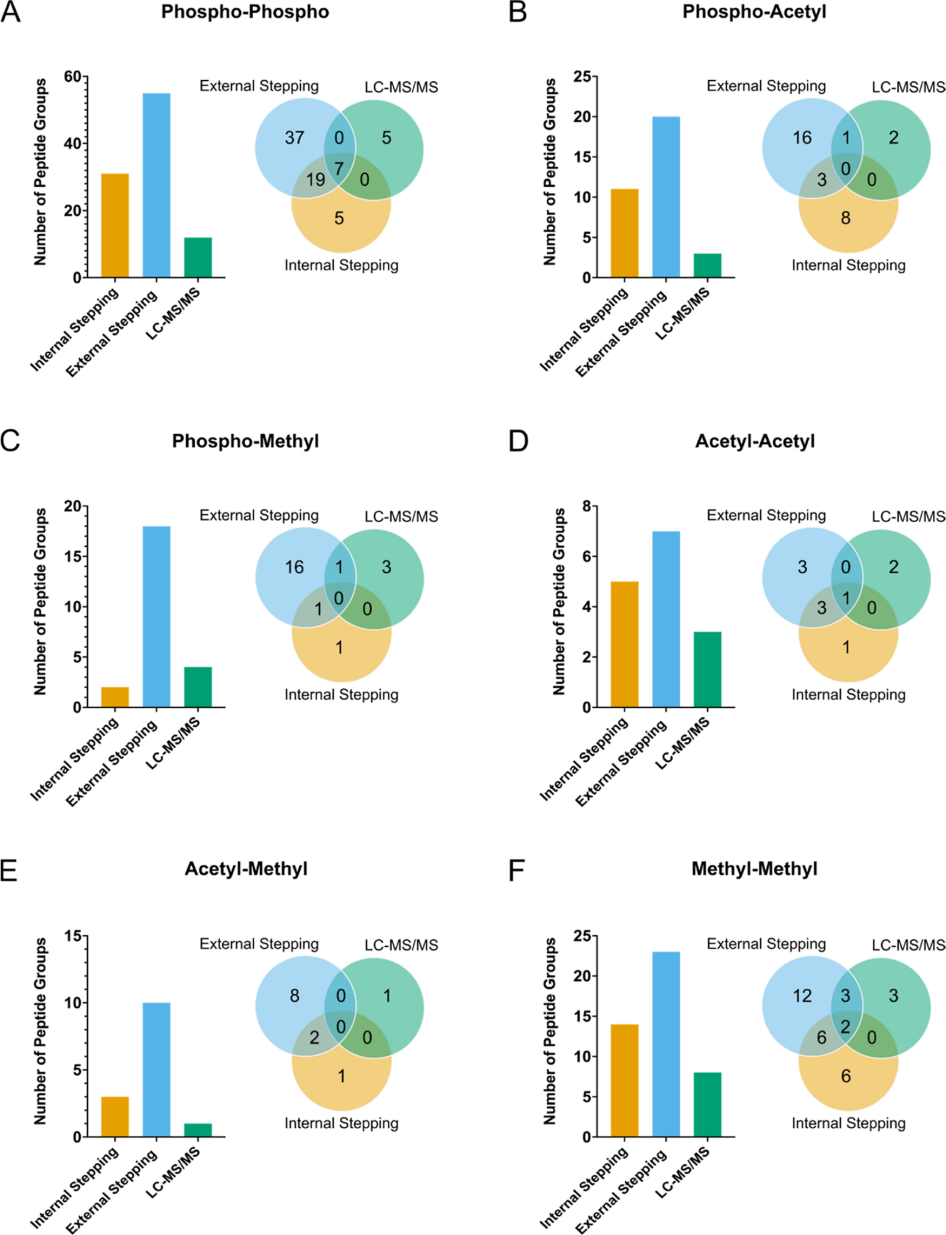
Internal stepping accelerates crosstalk identification rates compared to external stepping. The number of identified multi-PTM peptides versus FAIMS internal stepping, FAIMS external stepping, and standard LC-MS/MS for the PTM combinations: phospho–phospho (A), phospho–acetyl (B), phospho–methyl (C), acetyl–acetyl (D), acetyl–methyl (E), and methyl–methyl (F). Inserted Venn diagrams show overlapping peptides detected across multiple conditions.

Although significant for the identification of candidate positive PTM crosstalk sites, performing LC-MS/MS runs at static compensation voltages is time consuming; in our case, adding 3 h of run time per static compensation voltage. Thus, it could be debated whether the additional benefits outweigh the instrument time and costs. Thus, we next set up an internal stepping procedure whereby within a single LC-FAIMS-MS/MS run (3 h), the FAIMS compensation voltage cycled between −45, −60, −75, and −90 V. By comparing the LC-MS/MS results without FAIMS to internal stepping with the online FAIMS device incorporated into the workflow, the advantages of FAIMS can be realized without the drawback of additional instrument time. Figure 3 shows the comparison between the numbers of peptide groups identified for each condition. For all PTM combinations, the number of identified peptide groups increased 2-fold versus standard LC-MS/MS with the addition of FAIMS separation throughout the LC-MS/MS run (Figure 3e). Intriguingly, not all of the peptides identified in LC-MS/MS without FAIMS were identified in the internal stepping FAIMS condition. The reason for this is unclear, however, likely reflects the differing frequency of MS/MS events when FAIMS is applied. Nevertheless, internal stepping is advantageous over LC-MS/MS alone.

Comparing the sum of all of the static FAIMS compensation voltages with internal stepping involving four compensation voltages, the number of multi-PTM identifications was higher for external stepping for the majority of the PTM combinations searched (Figure 3). In total, external stepping LC-FAIMS-MS/MS identified 4 times more multi-PTM peptides containing the PTM phosphorylation, methylation, and acetylation than standard LC-MS/MS and 2 times more than internal stepping LC-FAIMS-MS/MS (Figure S3). This was expected due to the additional compensation voltages offering preferential separation of certain peptides. Thus, if time is unlimited, using a wide variety of compensation voltages is preferred.

### Expanding the Repertoire of Candidate PTM Crosstalk Sites

**Figure 4:**
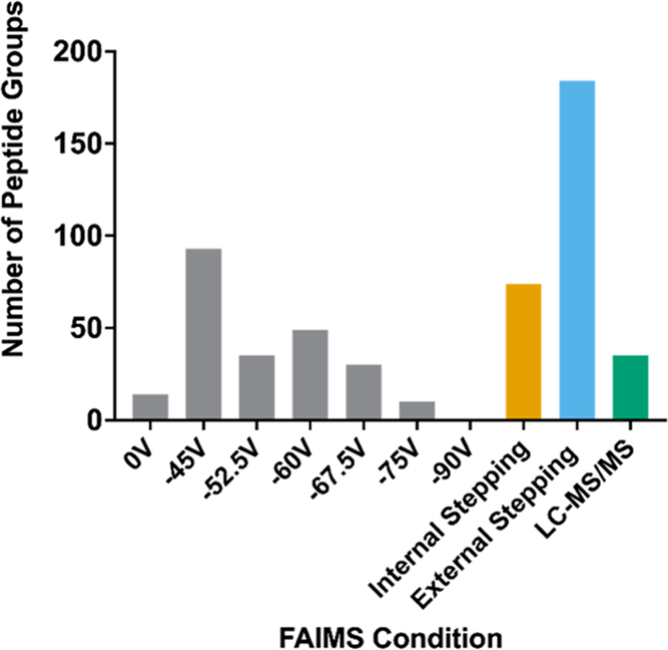
Expanding the repertoire of PTMs shows that more candidate PTM crosstalk sites are identified. The number of identified multi-PTM peptides for different FAIMS conditions including internal stepping (orange) and external stepping (blue) compared to standard LC-MS/MS (green). The PTM combinations included in the search were phosphorylation (S, T, Y), deamidation (N), nitrosylation (C, Y), acetylation (K, R, N-terminal), methylation (K, R), ubiquitinylation (K), and HexNAc (S, T, N).

Looking only at phosphorylation, acetylation, and methylation combinations, 180 candidate positive PTM crosstalk sites were identified using our LC-FAIMS-MS/MS approach. However, many other PTMs exist, with the potential for PTM crosstalk to occur between any of these PTM combinations. Thus, we next investigated whether our LC-FAIMS-MS/MS workflow could enhance the detection and identification of lesser-known yet equally important candidate crosstalk sites. Fifteen of the most common PTMs including nitrosylation, deamidation, ubiquitinylation, and HexNAc modifications were selected, and the number of multi-PTM-containing peptide groups identified using the search engine PMi-Byonic^29^ compared between standard LC-MS/MS and LC-FAIMS-MS/MS using the compensation voltages: 0, −45.0, −52.5, −60.0, −67.5, −75.0, and −90.0 V. To minimize the number of false-positive multi-PTM-containing peptides reported, a bespoke protein database was created, whereby only the proteins identified in two out of three replicates of our LC-MS/MS runs were included. Additionally, strict identification criteria were used, whereby the FDR was set to 0.01, a Byonic score cutoff of ≥300 applied, and 100% site localization was set for at least one PTM. Figure 4 shows the total number of multi-PTM-containing peptide groups identified for each FAIMS condition. Again, the addition of FAIMS to the standard proteomics workflow showed enhanced levels of the number of multi-PTM-containing peptides identified, and thus potential positive PTM crosstalk candidates demonstrating the benefit FAIMS has in enhancing the detection of any PTM-containing peptides. Certain multi-PTM-containing peptides are enriched at specific FAIMS compensation voltages (Table S1). Though the rationale for this is not clear in all cases, this data can serve as a good starting point for the selection of internal stepping compensation voltages when searching for specific PTM combinations. Moreover, it is important to note that since a restricted database was used and stringent filtering criteria, these results underestimate the number of multi-PTM-containing proteins present in the sample and thus the extent that PTM crosstalk is occurring.

### Biological Relevance of Identified Candidate Crosstalk Sites

**Figure 5:**
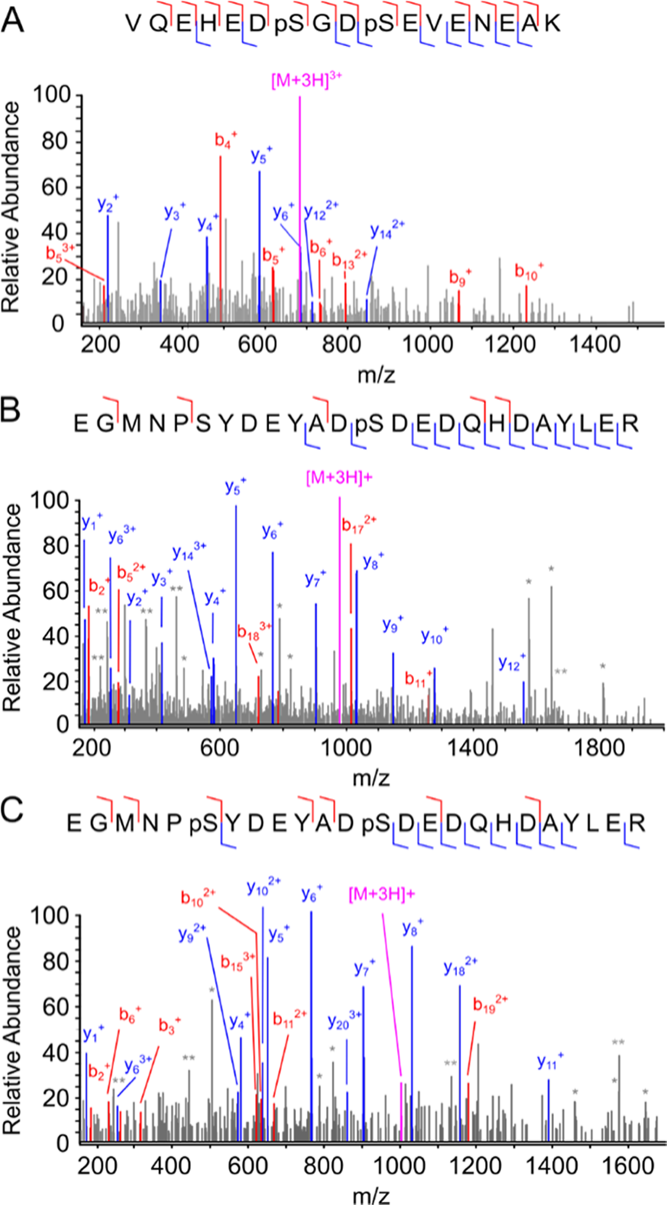
Examples of candidate PTM crosstalk sites. MS/MS spectra for double phosphorylated U3 small nucleolar RNA-associated protein 18 homologue peptide residues 115–131 (A) and FACT complex subunit SSRP1 residues 432–455 (B,C). The most abundant b ions (red) and y ions (blue) are highlighted on the mass spectra with all of the b and y ions detected corresponding to the respective peptides shown as colored lines on the inserted sequence. The [M + 3H]^3+^ (687.9 *m*/*z*), [M + 3H]^3+^ (977.0 *m*/*z*), and [M + 3H]^3+^ (1003.7 *m*/*z*) precursors were selected for fragmentation in parts (A)–(C), respectively. p indicates phosphorylation. Peaks marked * and ** represent b and y ions with phosphate loss and internal fragment ions, respectively.

In total, we have identified 398 candidate positive PTM crosstalk sites. Of these, 239 have previously been identified in either iPTMnet,^31^ PhosphoSitePlus,^32^ Proteomics DB,^33^ GlyGen,^34^ and dbSNO,^35^ validating our methodology. Due to the enrichment techniques available,^36^ hyperphosphorylation is the most commonly identified crosstalk within proteomics data sets. One example of this from our data is Src substrate cortactin, whereby two phosphorylation sites were detected, one four residues downstream of the other (pTPPVpSP), both of which have been reported previously and their kinases are known.^37−39^ Another example is the U3 small nucleolar RNA-associated protein 18 homologue, whereby Ser121 and Ser124 are phosphorylated (Figure 5a), phosphorylation sites that have previously been detected in multiple phosphoproteomics data sets.^31^ Another hyperphosphorylated protein of importance that was detected is nuclear ubiquitous casein and cyclin-dependent kinase substrate 1 (NUCKS), a protein whose phosphorylation status regulates DNA damage response^40^ and is proposed as a cancer biomarker.^41^ Indeed, both NUCKS and the previously mentioned protein Src substrate cortactin are themselves kinase substrates, thus a complex level of PTM crosstalk is likely prevalent.

When candidate PTM crosstalk sites are identified and their biological relevance is being considered, it is important to note the PTM site localization scores.^42^ If low-resolution MS2 data has been acquired, one should be aware of misidentification resulting from the high *m*/*z* tolerance when assigning spectra. Examples of this include the misidentification of one PTM for another PTM with a similar mass or when a combination of PTMs imparts a mass difference that also corresponds to two amino acids.^43^ Further to this, false localization rate has been shown to be particularly prevalent within the databases PhosphoSitePlus and PeptideAtlas^44^ so care needs to be taken to evaluate the evidence behind computationally searched MS2 data before commencing follow-up biological studies. In our workflow, since no enrichment steps are performed prior to peptide analysis, we frequently observe candidate PTM crosstalk sites, whereby the same peptide is also present harboring a single PTM. This provides additional evidence for PTM crosstalk and reduces false localization rates at these sites. For example, two peptides from the FACT complex subunit SSRP1 were observed; one with a single phosphorylated residue at Ser444 (Figure 5b) and another with two phosphorylated sites at Ser437 and Ser444 (Figure 5c). However, it is important to note that although these site localization scores are high, coisolation of a Tyr441 phosphorylated peptide or other noncanonical phosphorylation sites during MS1 cannot be ruled out. Thus, further work is always essential to validate whether the presence of these different combinations of these PTMs *in vivo* regulates its overall function.

## Conclusions

Crosstalk between PTMs has emerged as a highly relevant mechanism of protein regulation in signaling pathways, with aberrant crosstalk being implicated in disease. Currently, the upregulation of one PTM followed by western blotting and/or quantitative proteomics are widely used techniques for investigating PTM crosstalk. However, these studies lack information on which specific PTM is influencing another, information that is critical to ensure drugs can be designed to target the first critical incorrect switch that leads to disease. Moreover, putative positive PTM crosstalk sites whereby two PTMs are detected in close proximity within a protein sequence are challenging to detect by proteomics methods due to their low stoichiometry, a problem that is amplified with an increasing number of PTM sites.

Here, we designed an enrichment-free LC-FAIMS-MS/MS method to enhance the detection of candidate positive PTM crosstalk sites. We reasoned that FAIMS would separate peptides in an orthogonal manner to liquid chromatography, consequently improving the number of low-stoichiometry multiple-PTM peptides that would be selected for MS/MS. Indeed, LC-FAIMS-MS/MS identified 376 multi-PTM peptides compared to 66 that were identified using standard LC-MS/MS. This represents a 6-fold increase in multi-PTM-containing peptide identifications from a standard cancer cell line protein digest relative to standard LC-MS/MS. By cycling through different FAIMS compensation voltages during a single LC-FAIMS-MS/MS run, we were able to additionally increase candidate crosstalk sites identifications without compromising time and cost. Further to this, 40% of the identified candidate crosstalk sites have, to our knowledge, not previously been reported, with 83% of these uniquely identified with LC-FAIMS-MS/MS.

While innovative in its findings, further work is still needed to validate the candidate positive crosstalk sites reported. Moreover, it should be noted that MS2 fragmentation was performed in the ion trap to demonstrate the full capabilities of the Tribrid Orbitrap instruments in their ability to enhance duty cycle and thus peptide identifications. However, this inevitably also enhances false-positive identifications due to the low mass accuracy of ion trap analyzers used, a factor that is particularly relevant when performing MS2 on high charge state precursor ions. Low-resolution tandem MS analysis could also facilitate PTM misassignment due to the small mass differences between some PTMs.^43^ For example, lysine trimethylation could be misinterpreted as lysine acetylation—a difference in delta mass that corresponds to around 36 ppm. Thus, a balance needs to be struck between enhanced duty cycle and MS2 mass accuracy, and this needs to be carefully considered when performing experiments of this nature. We encourage manual validation of the MS/MS spectra when low mass accuracy is provided, and if uncertainties are present, high-resolution MS2 data acquired before additional biological experiments are performed. Additional modifications to the protocol could enhance further the detection of candidate positive crosstalk sites. For instance, we noticed that the candidate crosstalk sites we observe are longer than unmodified peptides (Figure S4). Thus, we anticipate that alternative enzymes that generate longer peptide sequences may be beneficial for enhancing the identification of candidate crosstalk sites. Further to this, our phosphorylation-based searches only accounted for phosphorylation of serine, threonine, and tyrosine residues. With mounting evidence relating to the significance of noncanonical phosphorylation at histidine, arginine, lysine, aspartate, glutamate, and cysteine residues,^45^ we believe that the addition of FAIMS to the standard LC-MS/MS workflow could also enhance the identification of noncanonical phosphorylation sites providing careful control of fragmentation techniques that are incorporated into the workflow and enhanced MS2 resolution through the use of the Orbitrap to ensure highly accurate PTM site localization.

In summary, our findings illustrate the potential of LC-FAIMS-MS/MS in expanding the repertoire of candidate PTM crosstalk sites that will not only enhance our understanding of how PTMs communicate with one another to alter protein function but also help us to find the critical switches between healthy and disease states that can offer new avenues for therapeutic intervention.

## Supporting Information Available

Figure S1: LC-FAIMS-MS/MS improves peptide and protein identifications relative to standard LC-MS/MS; Figure S2: Both internal and external stepping improves identification of multi-PTM peptides compared to standard LC-MS/MS; Figure S3: LC-FAIMS-MS/MS improves overall detection of candidate crosstalk sites compared to LC-MS/MS alone when searching pairwise for PTM combinations; Figure S4: Multiple-PTM-containing peptides are longer in sequence than the unmodified peptides identified in this study; and Table S1: Peptide groups detected in PTM-wide search using PMi-Byonic search with many PTMs (PDF)
Table S2: Multi-PTM-containing peptides identified in two out of three LC-(FAIMS)-MS/MS conditions (XLSX)

## Notes

The mass spectrometry proteomics data have been deposited to the ProteomeXchange Consortium via the PRIDE^46^ partner repository with the data set identifier PXD026448 and null.

## Abbreviations

PTM: post-translational modification
MS: mass spectrometry
LC: liquid chromatography
FAIMS: high-field asymmetric ion mobility spectrometry
