# Supplementary material for: FAIMS Enhances the Detection of PTM Crosstalk Sites
Source: J Proteome Res. 2022 Mar 2;21(4):930–9. doi: 10.1021/acs.jproteome.1c00721 (PMC8981314; doi:10.1021/acs.jproteome.1c00721)
Supplement: Supplementary file 1 — pr1c00721_si_001.pdf [file pr1c00721_si_001.pdf]

Supporting Information

FAIMS enhances the detection of PTM Crosstalk Sites

Kish R. Adoni[1], Debbie L. Cunningham[1], John K. Heath[1], Aneika C. Leney[1]*

[1]School of Biosciences, University of Birmingham, Edgbaston, Birmingham, B15 2TT, UK.

Table of Contents

Figure S1.docx: LC-FAIMS-MS/MS improves peptide and protein identifications relative to standard LC-MS/MS…………………………………………………………………..3

Figure S2.docx: Both internal and external stepping improves identification of multi-PTM peptides compared to standard LC-MS/MS………………………………………..….4

Figure S3.docx: LC-FAIMS-MS/MS improves overall detection of candidate crosstalk sites compared to LC-MS/MS alone when searching pairwise for PTM combinations…………….5

Figure S4.docx: Multiple PTM-containing peptides are longer in sequence than the unmodified peptides identified in this study…………………………………………………….……….6

Table S1. docx: Peptide Groups Detected in PTM-wide search using PMi-Byonic search with many PTMs…………………………………………………………………………………….7

Table S2: Multi-PTM containing peptides identified in two out of three LC-(FAIMS)-MS/MS conditions (xlsx).

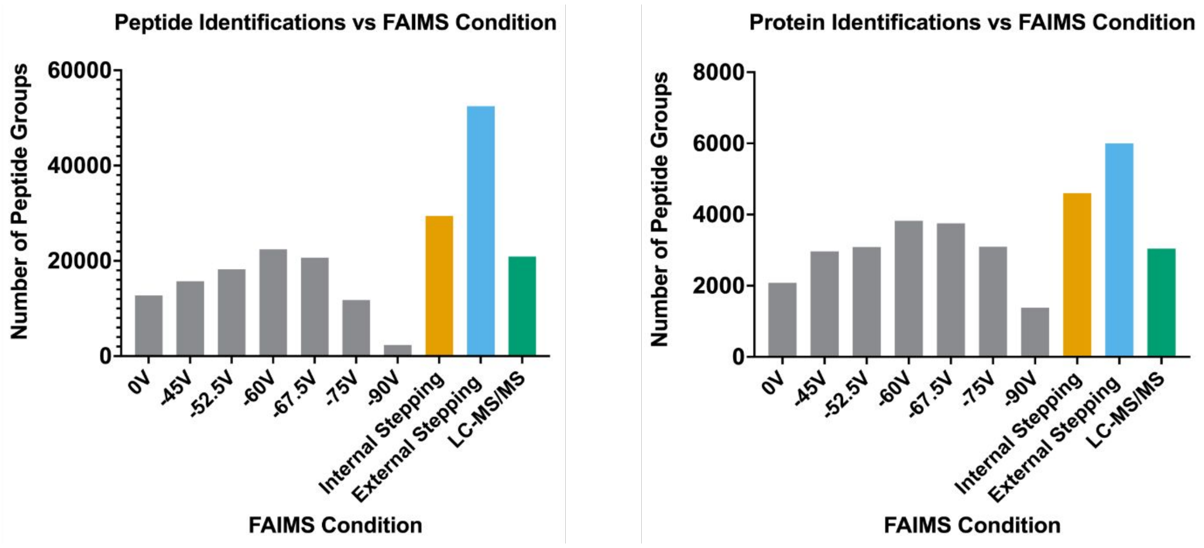

Figure S1: LC-FAIMS-MS/MS improves peptide and protein identifications relative to standard LC-MS/MS. (a) Number of peptide groups identified at different static compensation voltages as well as for internal stepping, external stepping and standard LC-MS/MS. (b) Number of proteins identified for same conditions as (a).

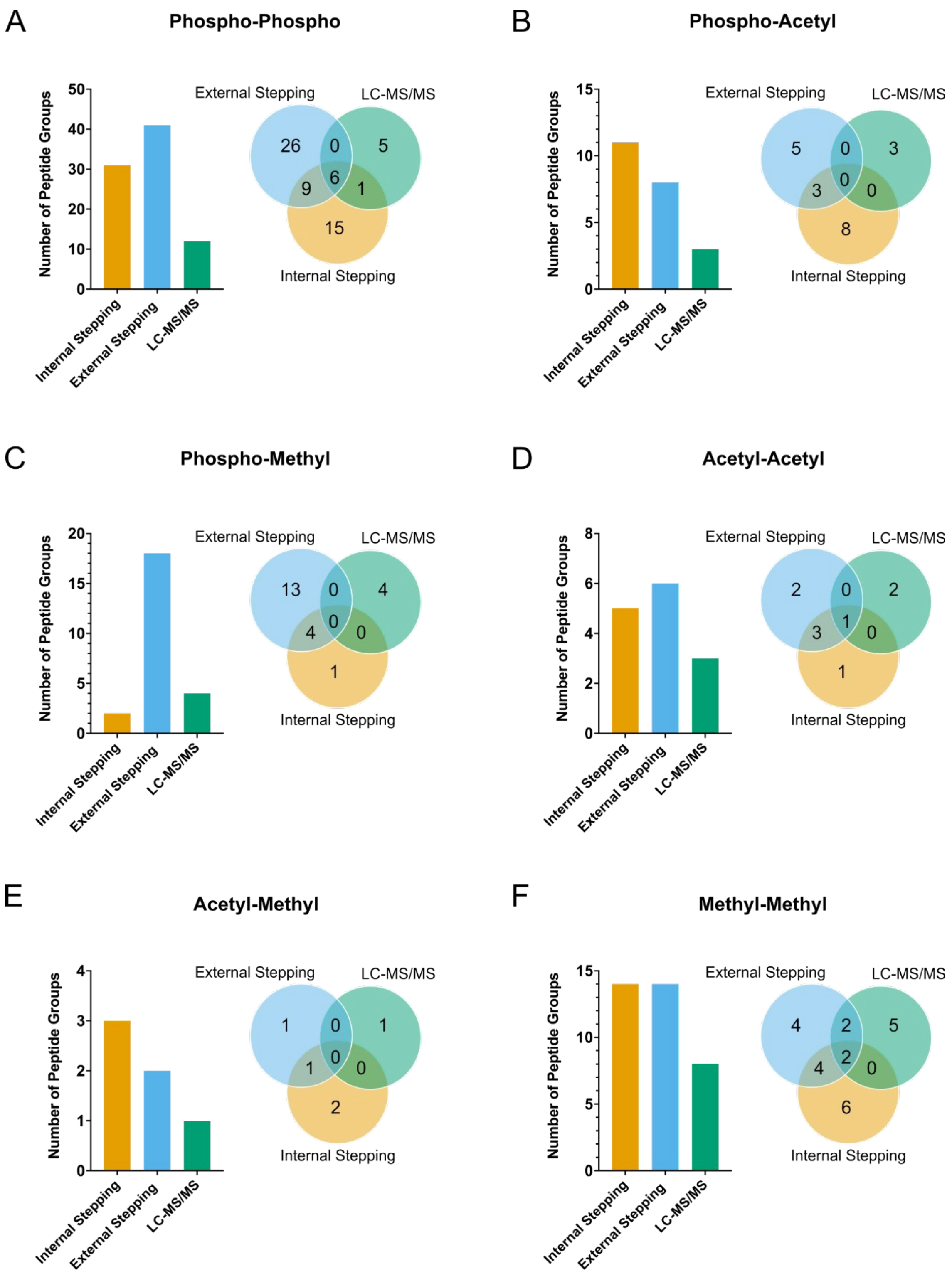

Figure S2: Both internal and external stepping improves identification of multi-PTM peptides compared to standard LC-MS/MS. The number of identified multi-PTM peptides versus internal stepping, external stepping (including only the identifications from static compensation voltages (CV): -45 V, -60 V, -75 V and -90 V equivalent to those used in

internal stepping) and standard LC-MS/MS for the PTM combinations: hyperphosphorylation (a), phosphorylation and acetylation (b), phosphorylation and methylation (c), hyperacetylation (d), acetylation and methylation (e), and hypermethylation (f).

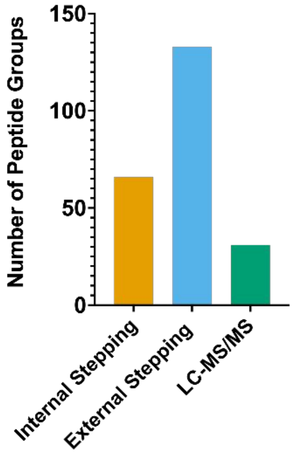

Figure S3: LC-FAIMS-MS/MS improves overall detection of candidate crosstalk sites compared to LC-MS/MS alone when searching pairwise for PTM combinations. The total number of multi-PTM containing peptides (peptide groups) detected using external stepping, internal stepping and standard LC-MS/MS.

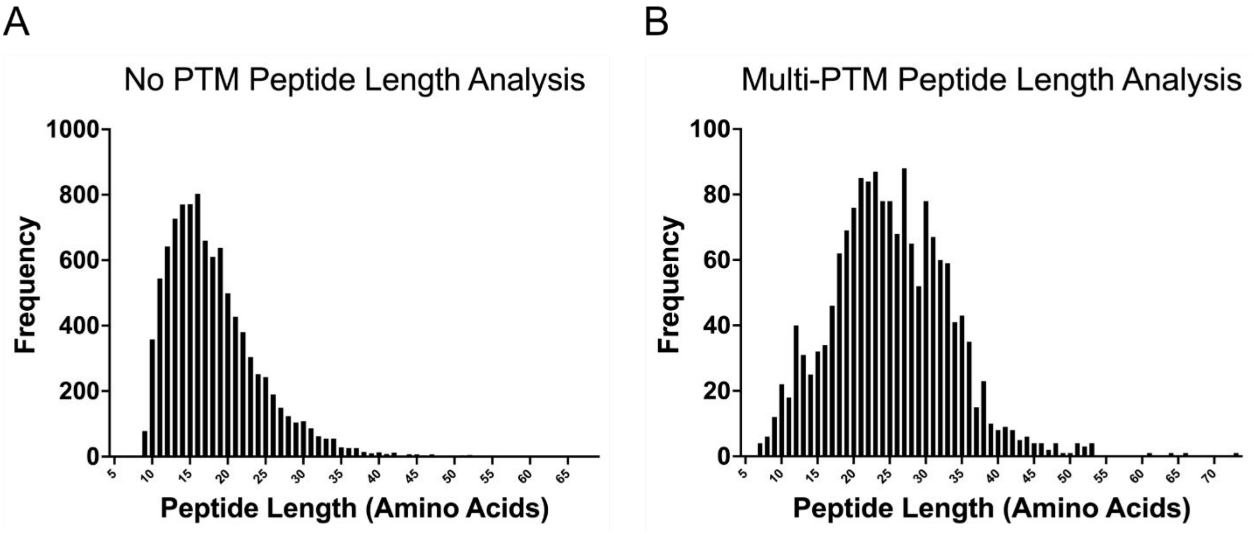

Figure S4: Multiple PTM-containing peptides are longer in sequence than the unmodified peptides identified in this study. To generate this data, all peptide group identifications within the study were filtered for high confidence peptides using an FDR of 0.01. The frequency of peptide groups that contained no modifications or a combination of two or more modifications were then plotted as a function of each sequence length from 6 amino acids upwards and the frequency plotted against peptide length.

Table S1: Peptide Groups Detected in PTM-wide search using PMi-Byonic search with many PTMs.

Multi-PTM combinations*	FAIMS Condition									
	0	-45	-52.5	-60	-67.5	-75	-90	Internal Stepping	External Stepping	LC-MS/MS
Multi-phosphorylation	6	8	5	6	2	-	-	9	27	7
Phosphorylation-acetylation	1	5	2	1	1	1	-	3	11	-
Phosphorylation-methylation	1	5	2	2	-	-	-	2	10	1
Phosphorylation-deamidation	8	13	8	10	5	1	-	6	45	9
Phosphorylation-HexNAc	-	3	-	-	-	-	-	-	3	-
Phosphorylation-ubiquitination	-	-	-	-	-	-	-	-	-	-
Phosphorylation-nitrosylation	-	7	1	-	-	-	-	-	8	1
Multi-acetylation	-	7	-	-	-	-	-	1	7	-
Acetylation-methylation	-	1	-	1	2	-	-	-	4	-
Acetylation-deamidation	3	-	1	3	2	1	-	6	10	-
Acetylation-HexNAc	-	1	-	-	-	-	-	-	1	-
Acetylation-ubiquitination	-	1	-	-	-	-	-	-	1	-
Acetylation-nitrosylation	-	2	-	1	2	1	-	2	6	-
Multi-methylation	10	6	8	11	3	1	-	24	39	8
Methylation-deamidation	6	10	6	11	3	1	-	5	2	37
Methylation-HexNAc	-	1	-	-	-	-	-	-	1	-
Methylation-ubiquitination	-	-	-	-	-	-	-	-	-	-
Methylation-nitrosylation	4	7	4	3	5	3	-	15	26	6
Multi-deamidation	-	2	-	2	-	-	-	1	4	-
Deamidation-HexNAc	-	-	1	-	-	-	-	-	1	-
Deamidation-ubiquitination	-	-	-	-	-	-	-	-	-	-
Deamidation-nitrosylation	1	3	1	-	2	-	-	-	7	1
Multi-HexNAc	1	-	1	-	-	-	-	-	2	-
HexNAc-ubiquitination	-	-	-	-	-	-	-	-	-	-

HexNAc-nitrosylation	-	3	-	-	-	-	-	-	3	-
Multi-ubiquitination	-	-	-	-	-	-	-	-	-	-
Ubiquitination-nitrosylation	-	1	-	-	-	-	-	-	1	-
Multi-nitrosylation	-	7	-	2	3	1	-	3	13	3
**Total candidate crosstalk sites**	**41**	**93**	**40**	**53**	**30**	**10**	**0**	**77**	**232**	**73**

* where multi-phosphorylation indicates any peptide group identified containing two or more phosphorylation sites, and phosphorylation-acetylation contains at least one of PTMs phosphorylation and acetylation. A dash indicates no peptides were detected.
